# Haematological Parameters and Spleen Rate of Asymptomatic and Malaria Negative Children in Edo South District, Nigeria

**DOI:** 10.5334/aogh.2458

**Published:** 2020-06-17

**Authors:** Damian Nwaneri, Olukayode Oladipo, Emeka Ifebi, Omoruyi Oviawe, Osaro Asemota, Bamidele Ogboghodo, Yetunde Israel-Aina, Ayebo Sadoh

**Affiliations:** 1 University of Benin/University of Benin Teaching Hospital, Benin City Nigeria, NG

## Abstract

**Background:**

Malaria is commonly associated with alteration in haematologic cells of infected individuals in both the acute uncomplicated and severe phases. Whether this alteration occurs in the asymptomatic phase of the disease is still being investigated.

**Objectives:**

To examine the haematocrit, thrombocytes, and monocytes levels of children with asymptomatic malaria compared with age/sex-matched controls who are malaria parasite negative and living in a stable malaria endemic region. It also set out to identify spleen rate of the children and to compare it with that observed in malaria negative controls.

**Methods:**

One hundred well-nourished children 2–9 years old with asymptomatic malaria parasitaemia and 100 age- and sex-matched malaria negative controls were recruited by multi-stage sampling from schools in a malaria endemic region of Nigeria. Malaria diagnosis was by microscopy, and each haematologic parameter was analysed following standard protocols.

**Results:**

Mean (±) monocyte count of 2.25 ± 0.9 × 10^9^ cells/L observed in asymptomatic malaria children was significantly higher than 1.34 ± 0.5 × 10^9^ cells/L observed in those with no malaria (*p* = 0.00). Mean (±) thrombocyte count was significantly lower (asymptomatic 203.64 ± 45.90 × 10^9^ cells/L Vs no malaria 230.91 ± 57.40 × 10^9^ cells/L) (*p* = 0.00). Spleen rate in the children was 15.5%. Presence of splenomegaly was not statistically significantly fewer in children with asymptomatic malaria parasitaemia (ASMP) (14/31) when compared to those who were malaria parasite negative (17/31) (χ^2^ = 0.34, *p* = 0.57). Similarly, there was no significant difference in the mean [±] spleen length of children with ASMP (n = 14; 2.86 ± 0.9 cm) and those who were malaria negative (n = 17; 2.53 ± 0.6 cm) (t = 1.22, *p* = 0.23).

**Conclusion:**

Thrombocytopaenia and monocytosis could be pointers to malaria parasitaemia in asymptomatic phase in a stable malaria region.

## Introduction

Malaria is a blood vector-borne disease with high morbidity and mortality in children in sub-Saharan Africa [1]. The 2017 malaria report of the World Health Organization (WHO) stated there were 216 million malaria cases worldwide in 2016, which was 5 million cases more than the 211 million observed in 2015 [1]. Malaria infection rate rises rapidly from 0% to 2% during the first three months of life, reaches 50% by the age of one1 year and then remains persistently high through the period of childhood in malaria stable endemic regions [1,2].

Malaria disease is commonly associated with alterations in haematologic cells of the peripheral blood of infected individuals in both the acute uncomplicated and severe phases [3,4]. The haematologic picture, however, varies from person to person and largely depends on nutritional status (parameters are severely depleted in malnourished than well-nourished children) [4], intensity of malaria transmission [4,5], age [3,6,7], and co-morbidities, such as helminthiasis [3].

Anaemia is a complication contributing significantly to mortality from malaria disease [3,4,5,6,7,8]. This usually results from excessive removal of non-parasitized erythrocytes, immune destruction of parasitized red cells, and impaired erythropoiesis by the bone marrow dysfunction [9]. Leukocytosis/monocytosis [4,10] or leucopaenia [4,5] and thrombocytopenia [4,5,9] have been documented in most severe acute disease [4,6]. The hypersplenism observed during acute malaria episodes is associated with splenomegaly and might also contribute to reduction in all the three blood cells causing anaemia, thrombocytopaenia, and leucopaenia [8]. The hypersplenism and malaria inflammatory response and endothelial activation have also been implicated as a cause of leukocytosis (monocytosis) [8,9]. The spleen is the vital organ for both malaria parasite clearance from the peripheral circulation and the observed immunological response to parasite clearance during the acute malaria episodes [8,9,11,12].

In most stable malaria endemic (high transmission) regions, the disease is largely asymptomatic [3,13,14]. It is not clear, however, whether asymptomatic malaria parasitaemia contributes to clinical malaria or confers protective immunity against malaria [3]. It has been documented that asymptomatic malaria parasitaemia (ASMP) serves as reservoir for malaria due to gametocyte transmission [13] and represents, perhaps, one step in the heterogeneous set of the disease pathways. The spleen rate, on the other hand, is another vital tool for evaluating malaria transmission in a given geographical location [14,15].

Although some studies had reported haematologic parameters of children with asymptomatic malaria, their findings were inconsistent. Most of these reports did not consider factors such as age/sex-match healthy population, nutritional status, and spleen rate of the study population.

This present study, therefore, reported haematologic parameters (haematocrit, thrombocytes, and monocytes) of well-nourished school children (i.e., children whose z-score for weight for age being within the 2-standard deviation for age/sex WHO growth chart) with ASMP and malaria parasite negative age- and sex-matched controls. The study also reported spleen rate in well-nourished school children, as well as comparing the spleen rate between children with ASMP and malaria negative age- and sex-matched controls.

### Research Questions

1. Are there differences in the levels of haematocrit, thrombocytes, and monocytes of well-nourished healthy children (2–9 years) with asymptomatic malaria and age/sex matched control with negative parasitaemia?
2. What is the relationship (if any) between these haematologic parameters and spleen rate in the study population?
3. Is there a correlation between the levels of these haematologic parameters and spleen length (size) in the study population?

### Null Hypothesis

There is no statistically significant difference in the levels of the haematologic parameters (haematocrit, thrombocytes, and monocytes) of well-nourished children with asymptomatic malaria parasitaemia and age/sex-matched healthy controls with negative parasitaemia. There is also no statistically significant correlation between in the level of these haematologic parameters and spleen size (length in cm) in the study population.

### Alternate Hypothesis

There is a statistically significant difference in the haematologic (haematocrit, thrombocytes, and monocytes) levels of well-nourished children with asymptomatic malaria parasitaemia and age/sex-matched healthy controls with negative parasitaemia. There is also a statistically significant correlation (either negative or positive) between the levels of these haematologic parameters and spleen size (length in cm) in the study population.

## Methodology

This was an observational analytic study carried out in school children aged 2–9 years recruited by multi-stage sampling in nursery and primary schools from 3 political wards in a local government located in Edo State Nigeria. The study location lies within the rain forest geographic region of Nigeria with an average annual temperature range of 27°C to 36°C, an annual rainfall of 3,500mm, and relative humidity of over 80.0% [17]. Participants’ recruitment and sampling was carried out between April and July 2017, which corresponds with high malaria transmission period (wet season). Prior to this study, malaria was said to be stable and holoendemic in the region [15].

Ethical clearance for the study was obtained from the Research and Ethics Committee of the University of Benin Teaching Hospital, Benin, while written permissions to access the schools were obtained from the Edo State Ministry of Education and the heads of the selected schools. Also, written informed consents were obtained from parents/guardians/caregivers of all selected participants.

### Subject Recruitment

Two hundred apparently healthy nursery and primary school pupils aged 2–9 years in Egor Edo South District Nigeria that satisfied the inclusion criteria for the study were recruited from the selected public and private schools. Inclusion criteria included (a) well-nourished children by the z-score for weight for age being within the 2-standard deviation for age/sex WHO growth chart, (b) the children would not have had treatment for malaria in the two weeks preceding the study, and (c) there is no history of fever at the time of recruitment. Excluded from the study were (a) children who were on antimalarial chemoprophylaxis during period of recruitment, as these could interfere with malaria parasites density; (b) children who had been diagnosed or had clinical features suggestive of sickle cell anaemia (SCA), SCA is a known cause of splenomegaly; (c) children who had received blood transfusion in the preceding three months. This is because the life span of red blood cells is three months to ensure that the assessed haematologic parameters were those of the index study participants.

Sample size calculation of 200 children based on effect size as outlined by Statistics Solutions at α = 0.05, β = 0.2 and a power of 0.80 [15]. These 200 children were then selected from the private and public primary schools by a multi-staged sampling technique as outlined below.

### Stage 1: Selection of Wards

There were 10 political wards in the selected local government area, namely Edaiken, Egor, Evbareke, Evhuotubu, Okhoro, Oliha, Ugbowo, Useh/Ogida, Uwelu, and Uselu. Three of the 10 wards were selected by simple random method using balloting technique. The three selected wards were Edaiken, Uwelu, and Ugbowo.

### Stage 2: Selection of Schools

There were 20 registered public and 10 registered private primary schools with nursery units in the three selected wards. Using the method proposed by Henderson et al., [16] one third (30%) of these schools were selected, giving a total of nine schools consisting of six public and three private schools. To select the specific schools, the names of the registered public and private primary schools were arranged on a list in alphabetical order. The first school was randomly selected from each list; subsequently, other schools were selected using a sampling interval obtained by dividing the total number of public or private primary/nursery schools in the selected wards on each list by the number of schools to be selected.

For the public schools, the sampling interval was calculated by dividing the total number of public primary schools in the 3 selected wards, which was 20, by the selected number of public primary schools, which was 6; equal to 3. The same was applicable with the private schools, where the sampling interval was calculated by dividing the total number of private primary schools in the 3 selected wards, which was 10, by the selected number of private primary schools, which was 3; giving 3.3, which was approximated to 3.

Therefore, from the 20 public schools in the 3 selected wards arranged in alphabetical order, the first school was selected by random technique, and thereafter, every other third school on the list was selected, and a total of 6 public schools were selected for the study. Similarly, 3 private schools were selected from the 10 registered private schools.

The selected public schools were labeled A, B, C, D, E, and F, and the selected private schools were labeled A1, B1, and C1.

### Stage 3: Determination of sample from each school

The total sample size of 200 was distributed proportionately among the selected public and private schools by the process shown below:

1 {{\rm{n}}_1} = \frac{{a\, \times \,c}}{b}

where: n_1_ = Sample size for each selected school

a = Population of pupils in index school

b = Total population of pupils in all selected schools

c = Calculated study sample size

The pupils were then selected from each school using the above formula until the entire sample size was reached.

### Stage 4: Determination of cohort sample size by age

The selected children in each school were stratified into age groups pre-school (2 to < 5 years) and school age (5–9 years). The specific age strata sample size was calculated for each school using the formula:

2 {{\rm{n}}_2} = \frac{{a\, \times \,c}}{b}

where: n_2_ = Age bracket sample size for each selected school

a = Population of pupils of index age bracket in index school

b = Total population of pupils in the index schools

c = Calculated sample size for index school

The class in each school from which the children were selected was determined by simple random sampling technique. The class register served as sampling frame from which each pupil was selected. Each selected pupil was given an informed consent form to be completed by their parents or caregiver. All selected pupils whose parents signed the informed consent were recruited into the study. In cases where a parent/guardian declined consent, the next child on the register was selected until the desired number was obtained. After obtaining a positive malaria positive result, the paired age group and sex matched negative in the same class was selected as control until the selected sample from that school was completed. This process was carried out in all the selected schools until the 100 children on both arms (malaria positive and malaria negative) were selected.

### Data collection and splenic assessment

At recruitment, a questionnaire developed by the authors, which was pre-tested on 20 pupils/parents from one of the schools that was not selected for the study, was administered to the parent/guardian of each child who fulfilled the selection criteria. Information on the questionnaire included data on socio-demographics, long-lasting insecticidal nets (LLIN) ownership and usage. A physical examination, including a general examination, measurement of temperature, anthropometry (weight and height), and abdominal examination were conducted and recorded in the study pro forma. The temperature of each pupil was measured using a clinical mercury-in-glass thermometer and the axillary temperature recorded in degrees Celsius after leaving the thermometer for a period of two minutes.

The anthropometric indices measured in the children was weight and height. Weight of the children was obtained as follows:

The participants were weighed using a Seca® Scale (Secagmbh & Co, Germany) with a precision to the nearest 0.1 kg. Each participant was weighed as follows:

- Shoes and outer clothing were removed.
- The scale was turned on by covering the solar panel for a second. When the number 0.0 appeared, the scale was ready.
- The child was asked to stand in the middle of the scale, feet slightly apart (on the footprints marked on the scale), and to remain still until the weight appeared on the display.
- The child’s weight was recorded to the nearest 0.1 kg.

The height of each child was taken using a standiometer with a precision to the nearest 0.1 cm. The height of each subject was measured as follows: each child was positioned erect, barefeet, with legs together, shoulders, buttocks, and heels touching the upright backboard and the child instructed to look straight ahead. The movable headpiece was brought onto the vertex of the head with sufficient pressure to compress the hair and the height was recorded to the nearest 0.1 cm.

The presence of splenic enlargement and the spleen length of all the children were assessed by bimanual palpation [17] by one of the authors who is a clinician. Bi-manual palpation has been observed to be useful and readily available for community-based studies [17]. Each child recruited in the study was instructed to lie down in the supine position on a couch provided by the researchers. The child was encouraged to relax the abdominal muscles by leaning forward slightly and breathing deeply. Subsequently, the tips of the fingers of the researcher’s right hand were pressed gently beneath the left costal margin and the participant was asked to take a long and deep breath as the palpation of a descending spleen was sought.

Blood samples were collected into containers with ethylene diamine tetra acetate (EDTA) anticoagulant using strict aseptic procedure. Each specimen bottle was labelled with each participant’s identification number as recorded on the questionnaire administered at recruitment. The venepuncture site was swabbed twice with methylated spirit and venous access gained with a 21G sterile hypodermic needle. One milliliter (1ml) of blood was withdrawn from each participant and transferred into the EDTA bottle to prevent clotting. Haemostasis was secured after the procedure by applying pressure to the venepuncture site with a clean dry swab. All samples for the day were transported to the Department of Child Health, UBTH Research Laboratory in an ice-packed polythene container with cork-screwed lid. The samples were used for preparation of the thick and thin smears for malaria parasite determination by the methods prescribed by Cheesbrough [18]. All films were prepared by one of the authors, who is a WHO-certified microscopist. The results were communicated to the parents/guardians/caregivers of the children as applicable. All children who tested positive for malaria parasite were given free treatment using the recommended arthemisinine-based combination therapy (ACT) [15]. Also, haemoglobin genotype was done for participants who had splenomegaly to rule out SCA.

Haematologic parameters (haematocrit, platelets, and monocytes) analyses were performed following standard protocols.

### Data Management

Data were generated from information (study questionnaire, physical examination findings, and laboratory results) obtained from each study participant. These were coded and analysed using the Statistical Package for Social Sciences (SPSS) version 21.0. (Chicago, IL, United State of America). There were two arms of the study participants, namely those with ASMP and those that were malaria parasite negative. These two groups were sex and age group matched. Households with more than five individuals were regarded as large households, while those with less than five individuals were regarded as small households [19]. The family social class was classified as lower, middle, and upper using the method described by Olusanya et al. [20] The haematologic parameters were classified using standard definitions, such as anaemia (haematocrit less than 30.0%), thrombocytopenia (platelet count less than 150 × 10^9^/L), and monocytosis (monocyte count more than 1.0 × 10^9^/L). Comparison of mean of quantitative variables, such as age and spleen length of the children and malaria parasite count, was done using independent t-test. Chi-square was used to test association between non-parametric variables, such as sex, family social class, household size, use of long-lasting insecticidal nets, and presence of malaria parasite and splenomegaly, respectively. Pearson’s correlation was used to identify the relationship between malaria parasitaemia count and spleen length. The level of significance for each variable was set at p < 0.05 and confidence level at 95%.

## Results

Of the 200 children (100 well-nourished children, 2–9 years old with ASMP and 100 age and sex matched controls with malaria parasite negative), mean (±) age was 6.6 ± 2.0 years. There were 59 (59.0%) males and 41 (41.0%) females in each arm of the study. Table 1 shows the demographic characteristics of the two groups of the study participants. In the ASMP group, the median parasite count was 2440.0 parasites/µL (range 560–4960 parasites/µL). *Plasmodium falciparum* was the only malaria parasite specie observed in the children.

**Table 1:** Demographic characteristics of the 200 study participants.

Socio-demographic characteristics	Asymptomatic malaria group	Control group	χ^2^	*p*-value

	n = 100	n = 100		

**Age (Years)**	19 (19.0)	19 (19.0)	0.00	1.00
2–< 5	81 (81.0)	81 (81.0)		
5–9				
**Sex**				
Male	41 (41.0)	41 (41.0)		
Female	59 (59.0)	59 (59.0)	0.00	1.00
**Family social class**				
Upper	10 (16.0)	2 (2.0)		
Middle	16 (16.0)	33 (33.0)	15.73	0.00*
Lower	82 (82.0)	57 (57.0)		
**Household size**				
Small	79 (79.0)	80 (80.0)		
Large	21 (21.0)	20 (20.0)	0.00	0.97

Total mean (±) haematocrit of the study population was 34.3 ± 2.2% (range 21–40%), mean (±) platelet count was 217.3 ± 53.6 × 10^9^ cells/L, and the mean (±) monocyte count was 1.80 ± 0.9 × 10^9^ cells/L.

Figures 1 A, B, and C show a box plot of the age of the children in relation to packed cell volume, monocyte, and thrombocyte counts. The packed cell volume of the children increased with age.

**Figure 1A:**
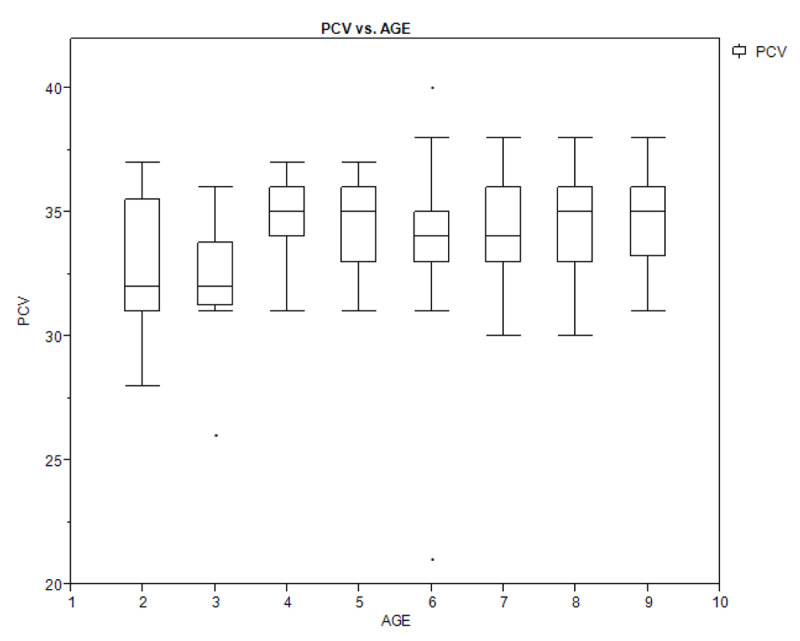
Box plot showing the relationship between age of the children and their packed cell volume.

**Figure 1B:**
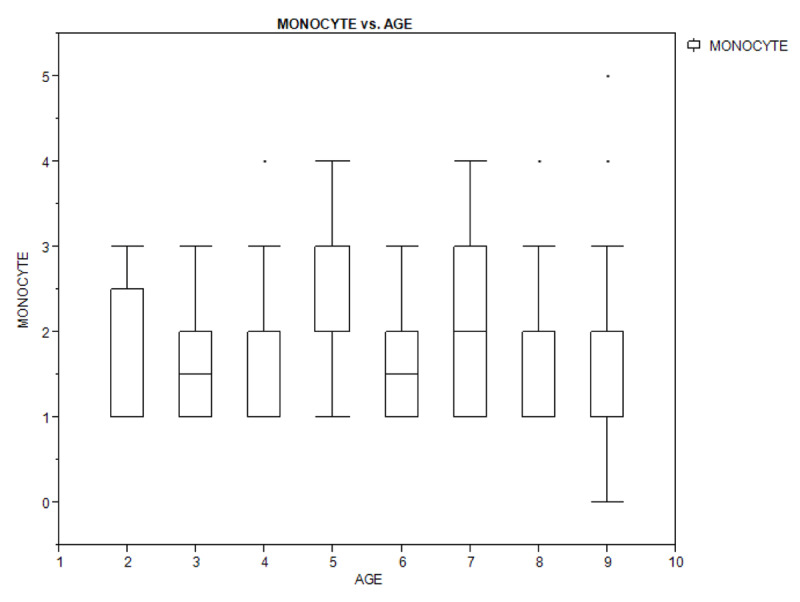
Box plot showing the relationship between age of the children and their monocyte count.

**Figure 1C:**
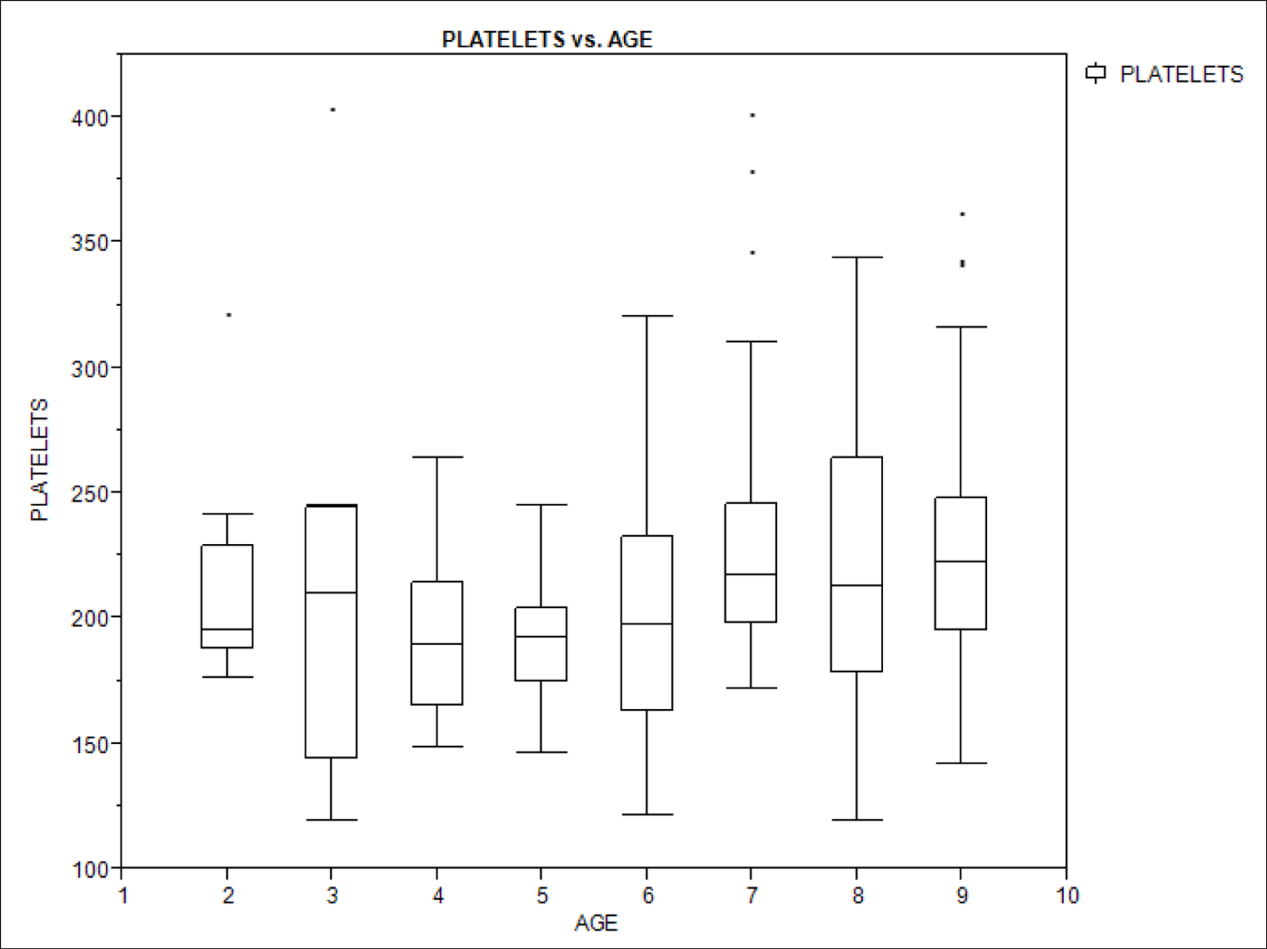
Box plot showing the relationship between age of the children and their platelets (thrombocyte) count.

Spleen rate observed in the children was 31/200 (15.5%). Mean spleen length of the study population was 2.7 ± 0.7 (2–5) cm. Presence of splenomegaly was not statistically significant in children with ASMP (14/31) when compared to those with malaria parasite negative (17/31) (χ^2^ = 0.34, *p* = 0.57). Similarly, there was no significant difference in the mean [±] spleen length of children with ASMP (n = 14; 2.86 ± 0.9 cm) and those with negative malaria parasite (n = 17; 2.53 ± 0.6 cm) (t = 1.22, *p* = 0.23). There was a positive correlation between MP count and spleen length; however, this positive correlation was not statistically significant (r = 0.22, *p* = 0.23).

Table 2 shows the haematologic parameters in the study population (ASMP versus malaria negative controls) and in children with splenomegaly and those with no splenomegaly. The mean (±) thrombocyte count 203.64 ± 45.87 × 10^9^ cells/L of the children with ASMP was significantly lower than 230.91 ± 57.37 × 10^9^ cells/L observed in those with negative malaria parasite (*p* = 0.00). The mean (±) monocyte count was significantly higher (2.25 ± 0.95 × 10^9^cells/L in children with ASMP) than 1.34 ± 0.5 × 10^9^ cells/L observed in those with negative malaria parasite (*p* = 0.00). There was no significant difference in haematocrit levels between the two groups (34.42% vs 34.15%; *p* = 0.39). There was also no significant difference in the mean haematologic parameters in children with splenomegaly and those with no splenomegaly.

**Table 2:** Haematologic parameters of the 200 study participants (ASMP versus malaria negative controls) in children with splenomegaly and those with no splenomegaly.

Haematologic parameters	Subjects		Splenomegaly	

	Asymptomatic malaria group	Control group	Present	Absent

	n = 100	n = 100	n = 31	n = 169

**Haematocrit (%)**				
Mean	34.42	34.15	34.30	34.30
SD [±]	2.30	2.10	2.10	2.30
95% CL	–0.35, 0.89		–0.85, 0.86	
*p*-value	0.39		1.00	
**Thrombocyte count (×10^9^/L)**				
Mean	203.64	230.91	217.50	217.20
SD [±]	45.90	57.40	62.50	52.00
95% CL	–41.76, –12.79		–20.45, 20.94	
*p*-value	0.00*		0.98	
**Monocyte count (×10^9^/L)**				
Mean	2.25	1.34	1.81	1.79
SD [±]	0.90	0.50	1.01	0.90
95% CL	0.70, 1.12		–0.33, 0.35	
*p*-value	0.00*		0.94	

p-value* – significant.

Table 3 shows bivariate analysis of the haematologic parameters in the study population. The proportion of the children with ASMP who had thrombocytopaenia (13.0%) was significantly higher than the 2.0% observed in the control group (Fisher’s Exact Test; OR = 7.3, *p* = 0.003). The children with ASMP were 6.8 times likely to have thrombocytopaenia than the control group. Similarly, the proportion of children with ASMP who had monocytosis (77.0%) was significantly higher than the 33.0% observed in the control group, and those were nearly 7 times more likely to have monocytosis than the control group (χ^2^ = 39.11, OR = 6.8, *p* = 0.00). Although there was no significant difference in the haematocrit levels between the two groups, the children with ASMP were two times more likely to develop anaemia than the control group (Fisher’s Exact Test: OR = 2.0, *p* = 0.56).

**Table 3:** Bivariate analysis of the haematologic parameters of the 200 study participants (ASMP versus malaria negative controls).

Haematologic parameters	Subjects		χ^2^	OR	*p*-value

	ASMP	Control group			

	n = 100	n = 100			

**Haematocrit (%)**					
Anaemia	2 (2.0)	1 (1.0)	*	2.0	0.56
Normal haematocrit	98 (98.0)	99 (99.0)			
**Thrombocyte count (×10^9^/L)**					
Thrombocytopaenia	13 (13.0)	2 (2.0)	*	7.3	0.003
Normal thrombocyte count	87 (87.0)	98 (98.0)			
**Monocyte count (×10^9^/L)**					
Monocytosis	77 (77.0)	33 (33.0)	39.11	6.8	0.00
Normal monocyte	23 (23.0)	67 (67.0)			

* Fisher’s Exact Test.

Table 4 shows bivariate analysis of the haematologic parameters in the study population with regards to splenomegaly. There was no significant difference in the haematologic parameters of children with splenomegaly and those with no splenomegaly, and spleen length did not depend on the haematologic levels (packed cell volume, monocyte, and platelet counts) as shown by the box plot (Figures 2 A, B, and C).

**Table 4:** Bivariate analysis of the haematologic parameters of the 200 study participants (splenomegaly versus no splenomegaly).

Haematologic parameters	Splenomegaly		χ^2^	OR	*p*-value

	Present	Absent			

	n = 31 (%)	n = 169 (%)			

**Haematocrit (%)**					
Anaemia	0 (0.0)	3 (1.8)	*0.56	0.9	1.00
Normal haematocrit	31 (100.0)	166 (98.2)			
**Thrombocyte count (× 10^9^/L)**					
Thrombocytopaenia	1 (3.2)	14 (8.3)	*0.97	0.4	0.33
Normal thrombocyte count	30 (96.8)	155 (91.7)			
**Monocyte count (× 10^9^/L)**					
Monocytosis	17 (54.8)	93 (55.0)	0.00	1.0	1.00
Normal monocyte	14 (45.2)	76 (45.0)			

**Figure 2A:**
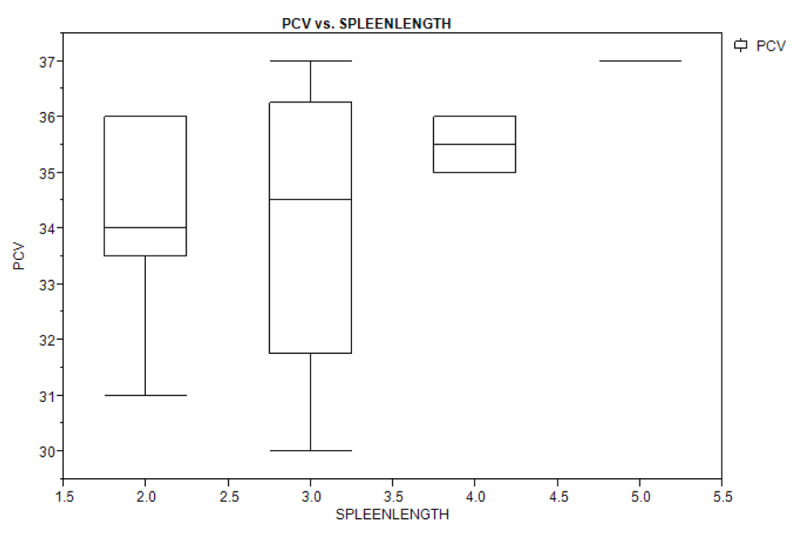
Box plot showing the relationship between spleen length and packed cell volume of the study population.

**Figure 2B:**
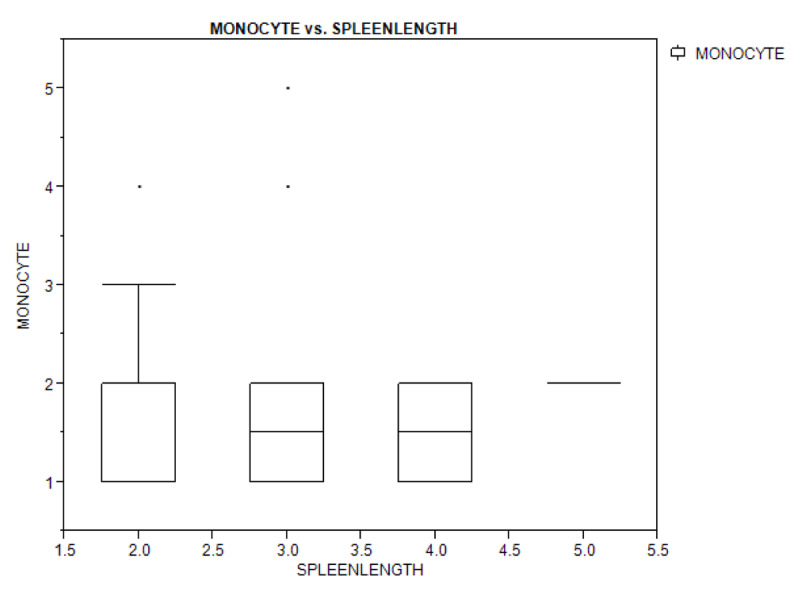
Box plot showing the relationship between spleen length and monocyte count of the study population.

**Figure 2C:**
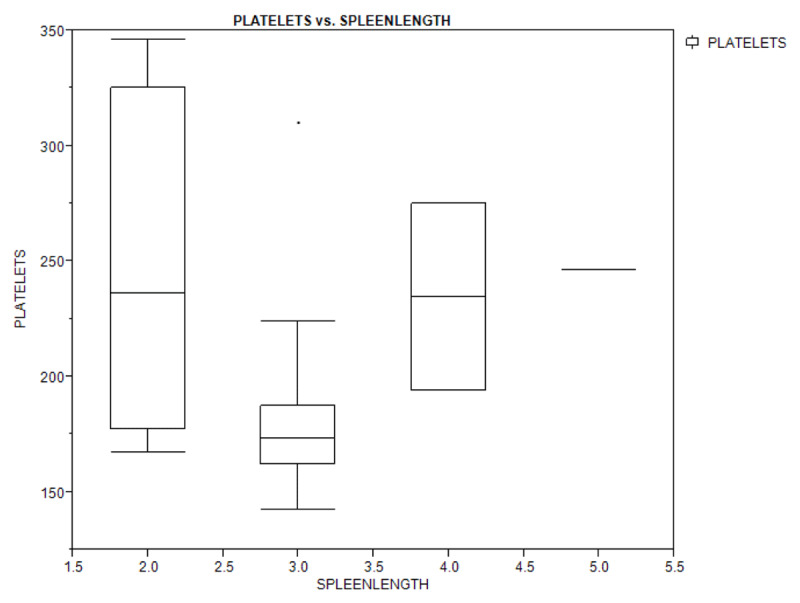
Box plot showing the relationship between spleen length and platelets count of the study population.

In the study population, 110 (55.0%) had one or more LLIN in their households and 73 used the LLIN as a means of malaria prevention, giving an LLIN usage rate of 66.4%, while 37 (33.6%) did not use LLIN. The proportion of the children who did not use LLIN (26/37; 70.3%) significantly had ASMP as against the proportion of children who used LLIN (31/73; 42.5%) (χ^2^ = 7.60, *p* = 0.01).

## Discussion

This study showed that children with ASMP had a significant reduction in platelet count and significant likelihood of having thrombocytopaenia. Although thrombocytopaenia has been documented in both the acute and asymptomatic phases of malaria disease, its mechanism of development has not been fully understood [4,5,6,8,9] Some authors had documented peripheral destruction and consumption of platelets during immune response against malaria parasites, platelet activation by immune complex formation, hyper-aggregation, and damage of surface contact of platelet as possible causes [4,8]. This finding corroborates that of Igbeneghu et al. [6] in an adult population in Southwestern Nigeria, where it has been recommended that thrombocytopaenia could be a useful tool to screen out blood donors with malaria parasiataemia. Another implication of thrombocytopaenia in ASMP is splenomegaly and identification of spleen rates. Some authors postulated thrombocytopaenia in malaria is due to immune response generated by malaria antigen against the host macrophages leading to sequestration of the platelets in the spleen contributing to splenomegaly [3]. Spleen rate is a veritable tool for classifying malaria transmission and endemicity. Spleen rate is defined as the proportion of persons of a defined age range in a given population who have enlarged spleen, expressed in percentages [15]. Spleen rate of 15.5% observed in this study had, therefore, re-classified malaria transmission in the study locale from holoendemic to mesoendemic according to the WHO classification of malaria endemicity [15,21].

Estimate of burden is currently based on ASMP, spleen rate, and anaemia. Anaemia has been documented as common morbidity in acute and chronic malaria disease. Inflammatory responses against malaria antigen had been found to suppress erythropoiesis, shortening the life span of the red blood cells by inducing haemolysis and increasing clearance of both parasitized and non-infected erythrocytes [3,8]. Other causes of anaemia include under-nutrition and inadequate dietary in-take of nutrients, especially iron [22]. This study attempted to control for under-nutrition by including only children with normal z-score weight for age in the study and control arms. There was no significant difference in the presence of anaemia in children with ASMP and the controls. The children with ASMP, however, were two times more likely to develop anaemia, especially in under-fives who are also the more vulnerable group to malaria infection than the older children. Based on the fact haematologic parameters observed in this study were not dependent on age and that confounders, such as effect of nutrition on anaemia, was accounted for during recruitment of the study participants, it is recommended that thrombocytopaenia and spleen rate be included as a malariometric index for identification of malaria intensity and transmission in an apparently healthy population (2–9 years).

The spleen rate observed in this study was lower than the 28.9% observed by Udoh et al. [14] in children aged 2–10 years in Calabar South-south region of Nigeria. The low spleen rate observed in this study could be attributed to an improved malaria control programme in the study location. The National Malaria Elimination Program (NMEP) has instituted a strategic plan to educate the public through a step-down Advocacy, Communication and Social Mobilization (ACSM) strategy from the federal level to the communities at the local government level on malaria control/preventive measures [23,24]. One such strategy is the public campaign on mass distribution and use of the LLIN at the community level for malaria vector control [23,24]. Although the LLIN ownership of 55.0% observed in this study falls short of what had been documented earlier by Nwaneri et al. [25] in the same study location, LLIN usage rate of 66.4% was higher than what has been documented by some authors elsewhere in Nigeria [25]. This shows a remarkable improvement on this malaria vector control strategic tool compared to previous findings.

Monocytosis observed in this study could be a hallmark of conferment of immunity by ASMP in children. Some authors had documented leukopaenia in a population with ASMP (depletion of the leucocytes in response to chronic malaria infection) [3]. Although this study did not follow up on the children with ASMP to know whether they would eventually develop malaria disease, the corresponding increase in immune cells, such as monocytes, could signify the immune response to remove malaria parasites from the parasitized red blood cells. The macrophages originating from monocytes have been documented to be responsible for innate non-specific immune response for removal of the malaria antigens and had been implicated in other inflammatory responses in malaria disease, such as endothelial cell damage and sequestrations of parasitized red blood cells and platelets in the spleen [3,8]. Some authors had also documented that these cells, including the poly morphonuclear neutrophils in vitro, are also capable of extracting *P. falciparum* parasites from red blood cells without destruction of the cells [8,26]. Monocytosis in both acute and asymptomatic phase of malaria infection have been documented by some authors, showing the significance of these haematological cells in malaria immunology and/or progression of disease [26].

### Limitation

The children could have been screened for helminthes, which is a known cause of monocytosis. ASMP children were treated using the recommended ACT; however, there is need to re-assess the haematologic parameters of these children after the treatment.

## Conclusion

This study has shown that there are alterations in haematocrit, platelet, and monocyte counts in ASMP. Using spleen rate, it could be concluded that malaria transmission in this study location is currently mesoendemic rather than holoendemic as had been documented in previous studies. Thrombocytopaenia and spleen rate could be included as malariometric indices in classifying malaria endemicity. Thrombocytopaenia and monocytosis could be useful parameters for identification of asymptomatic malaria parasitaemia in children.

## Data Accessibility Statement

The datasets used and analysed during the current study are available from the corresponding author on reasonable request.

## Abbreviations

ACSM – Advocacy Communication and Social Mobilization

ACT – Arthemisinine-based Combination Therapy

ASMP – Asymptomatic Malaria Parasitaemia

EDTA – Ethylene Diamine Tetra Acetate

ITN – Insecticide Treated Nets

LLIN – Long-lasting Insecticidal-treated Nets

NMEP – National Malaria Elimination Program

SPSS – Statistical Package for Social Sciences

UBTH – University of Benin Teaching Hospital

WHO – World Health Organization
